# Comparison of three different screw trajectories in osteoporotic vertebrae: a biomechanical investigation

**DOI:** 10.1186/s12891-021-04254-0

**Published:** 2021-05-05

**Authors:** J.-S. Jarvers, S. Schleifenbaum, C. Pfeifle, C. Oefner, M. Edel, N. von der Höh, C.-E. Heyde

**Affiliations:** 1 Department of Orthopedic Surgery, Traumatology and Plastic Surgery, Leipzig University, Liebigstraße 20, 04103 Leipzig, Germany; 2 ZESBO – Zentrum zur Erforschung der Stuetz- und Bewegungsorgane, Leipzig University, Semmelweisstraße 14, 04103 Leipzig, Germany

**Keywords:** CBT, Biomechanical analysis, Cement-augmented screws, MC, Osteoporosis, Patient-specific placement guide, TT

## Abstract

**Background:**

Pedicle screw insertion in osteoporotic patients is challenging. Achieving more screw-cortical bone purchase and invasiveness minimization, the cortical bone trajectory and the midline cortical techniques represent alternatives to traditional pedicle screws. This study compares the fatigue behavior and fixation strength of the cement-augmented traditional trajectory (TT), the cortical bone trajectory (CBT), and the midline cortical (MC).

**Methods:**

Ten human cadaveric spine specimens (L1 - L5) were examined. The average age was 86.3 ± 7.2 years. CT scans were provided for preoperative planning. CBT and MC were implanted by using the patient-specific 3D-printed placement guide (MySpine®, Medacta International), TT were implanted freehand. All ten cadaveric specimens were randomized to group A (CBT vs. MC) or group B (MC vs. TT). Each screw was loaded for 10,000 cycles. The failure criterion was doubling of the initial screw displacement resulting from the compressive force (60 N) at the first cycle, the stop criterion was a doubling of the initial screw displacement. After dynamic testing, screws were pulled out axially at 5 mm/min to determine their remaining fixation strength.

**Results:**

The mean pull-out forces did not differ significantly. Concerning the fatigue performance, only one out of ten MC of group A failed prematurely due to loosening after 1500 cycles (L3). Five CBT already loosened during the first 500 cycles. The mean displacement was always lower in the MC. In group B, all TT showed no signs of failure or loosening. Three MC failed already after 26 cycles, 1510 cycles or 2144 cycles. The TT showed always a lower mean displacement. In the subsequent pull-out tests, the remaining mean fixation strength of the MC (449.6 ± 298.9 N) was slightly higher compared to the mean pull-out force of the CBT (401.2 ± 261.4 N). However, MC (714.5 ± 488.0 N) were inferior to TT (990.2 ± 451.9 N).

**Conclusion:**

The current study demonstrated that cement-augmented TT have the best fatigue and pull-out characteristics in osteoporotic lumbar vertebrae, followed by the MC and CBT. MC represent a promising alternative in osteoporotic bone if cement augmentation should be avoided. Using the patient-specific placement guide contributes to the improvement of screws’ biomechanical properties.

**Supplementary Information:**

The online version contains supplementary material available at 10.1186/s12891-021-04254-0.

## Background

Lumbar spine instrumentation using pedicle screws has emerged as the most common surgical technique in traumatic and degenerative surgery. Especially in the case of treating medical indications caused by osteoporosis, cement-augmented cannulated pedicle screws are widely used by now [13, 23, 32]. As bone cement, e.g. polymethylmethacrylate (PMMA), is associated with inherent disadvantages [17, 25, 38], auspicious alternatives are needed to achieve sufficient stability. To overcome this issue by enhancing pedicle screw fixation in bone of compromised quality, different screw designs and insertion techniques regarding screw trajectory modifications were developed [36, 40]. In 2009, Santoni et al. [35] introduced the cortical bone trajectory (CBT) fixation approach. This trajectory starts medially at the pars interarticularis and follows a craniolaterally direct path through the pedicle [26]. By contrast, the medially directed traditional trajectory (TT) has a lateral starting point and uses a transpedicular path through the anatomic axis of the pedicle [29]. Accordingly, TT pedicle screws achieve their stability apart from the pedicle mainly in cancellous bone, which is why a loss of stability can be seen in osteoporotic patients if no bone cement is used. In contrast, CBT screws are characterized by increased screw thread contact with cortical bone [26]. A further alternative represents the midline cortical (MC) approach, which is derived from the CBT technique. Its entry points are sufficiently distant from the adjacent facet joints. The trajectory follows the path from the pars interarticularis to the inferior edge of the pedicle [29]. Due to passing denser bone, the insertion of longer screws, which are directed towards the middle of the vertebral endplate, is possible. Although CBT techniques allow invasiveness reduction and show comparable or superior biomechanical features compared to the TT approach [6, 14, 18], there is little consensus on the selection of the optimal screw size and the corresponding screw path [30]. While the original CBT method grants a screw length usually no longer than 25–30 mm, the MC approach allows the use of longer screws with a minimum length of 40 mm. However, previous literature does not adequately address the effects of the different insertion techniques on the screws’ biomechanical performance. For that reason, this study aimed at comparing the fatigue behavior and fixation strength of pedicle screws using the cement-augmented TT, the CBT, and the MC fixation approach, respectively. Therefore, a biomechanical analysis was performed to evaluate whether CBT screws or MC screws represent a possible alternative to cement-augmented TT screws.

## Methods

### Specimens and grouping

Ten adult human cadaveric spine specimens, especially L1 to L5, without destructive pathologies (fractures, tumor) were obtained in fresh and anatomically unfixed condition. All donors originated from the Institute of Anatomy of the Leipzig University and had given written consent to dedicate their bodies to medical education and research purposes. Being part of the body donor program regulated by the Saxonian Death and Funeral Act of 1994 (3rd section, paragraph 18, item 8), institutional approval for the use of the post-mortem tissues of human body donors was obtained. The authors declare that all experiments were performed according to the ethical principles of the Declaration of Helsinki.

During dissection, all vertebrae were separated into single levels. Muscular and soft tissue was removed from each vertebra while preserving its anatomy. The specimens were stored at − 83 °C until testing. Bone mineral density (BMD) was measured by dual-energy X-ray absorptiometry (DXA) using Hologic Delphi A QDR-Series (Hologic, Inc., Marlborough, MA, USA). For this purpose, spine (L1 to L5) without tissue was analysed and the average BMD of each vertebra of the corresponding specimen was calculated. In addition, a low-dose computed tomography (CT) scan (PHILIPS Brilliance iCT 256, Philips Healthcare, Cleveland, OH, USA) of all specimens was taken for the exclusion of bone defects and for preoperative planning.

All cadaveric spine specimens (*n* = 10) were randomized to two different groups, each consisting of 20 lumbar vertebrae (L1 - L4) from five body donors. While group A analysed CBT screws vs. MC screws, group B tested MC screws vs. cement-augmented TT screws. Additionally, both groups were divided into the same two subgroups concerning the test procedure (dynamic test, static test). As L2 and L4 were tested dynamically, L1 and L3 were analysed under static testing conditions. Moreover, in group B four more L5 were tested.

### Preoperative planning

**Fig. 1:**
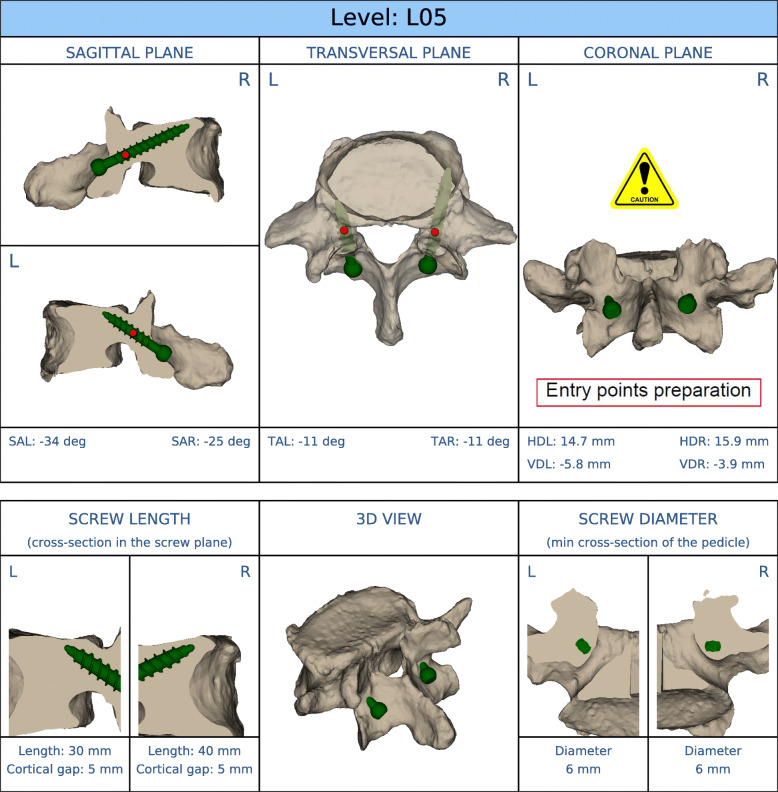
Preoperative 3D planning of screw design and appropriate screw trajectory (SolidWorks® 3D CAD software (Dassault Systèmes SolidWorks Corporation, Vélizy Villacoublay, France)

Low-dose CT scans of all cadaveric specimens were taken. While the TT screw design was determined by an experienced surgeon by using these scans, the planning of the CBT screws and MC screws was more extensive. First, a CT scan-based 3D model of every single vertebra was reconstructed by using medical image processing software Mimics® (Materialise NV, Leuven, Belgium). A 3D preoperative plan regarding the optimal screw design (screw length, screw diameter) and screw trajectory (sagittal, transverse, and coronal plane) was realized using SolidWorks® 3D CAD software (Dassault Systèmes SolidWorks Corporation, Vélizy-Villacoublay, France) (Fig. 1) Subsequently, the MySpine® patient-matched targeting guide was generated based on these data. Relevant expertise and equipment originated from Medacta International SA (Castel San Pietro, Switzerland).

### Test preparation

**Fig. 2:**
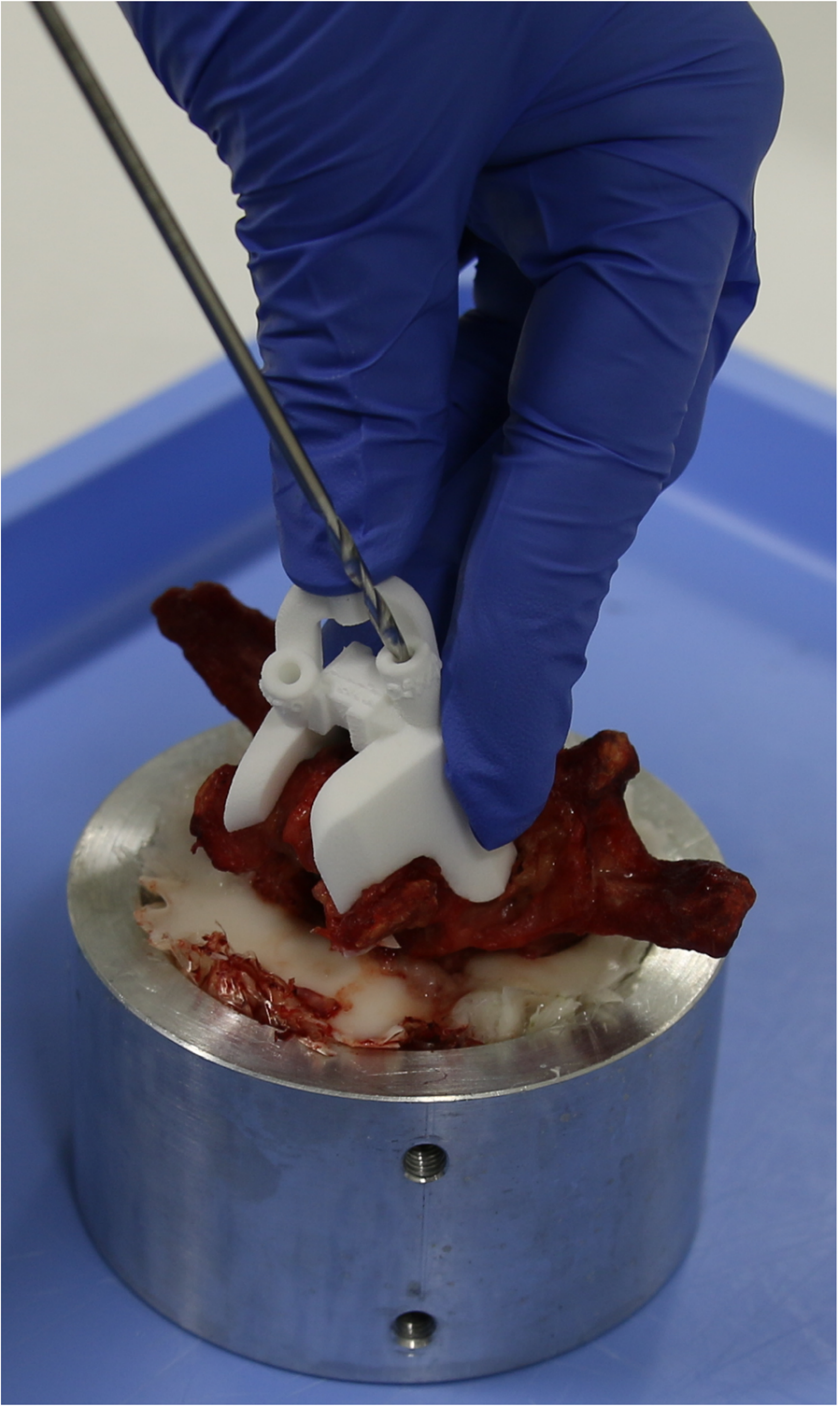
Guided drilling of screw trajectory

**Fig. 3:**
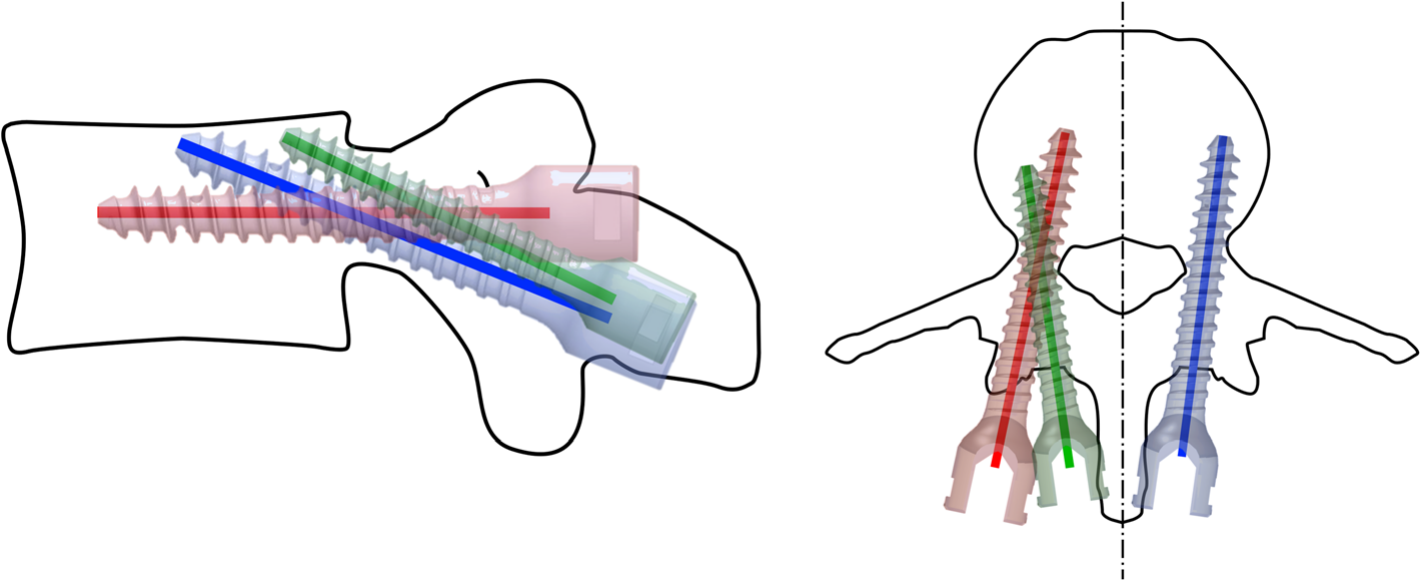
Screw path of traditional trajectory (red), cortical bone trajectory (green) and midline cortical (blue) fixation approach, respectively; left: sagittal plane, right: transverse plane

Before biomechanical testing, the lumbar vertebra was thawed to room temperature for 24 h and embedded in an aluminum cylinder by using RenCast® FC52/53 Isocyanate mixed in a ratio of 1:1:3 with RenCast® FC52 Polyol and Filler DT 082 (Huntsman Corporation, Salt Lake City, UT, USA). After finalizing preparations, all vertebrae were instrumented by the same experienced surgeon. For screw implantation, the patient-specific placement guide (MySpine®, Medacta International SA, Castel San Pietro, Switzerland [9, 15, 22]), (Fig. 2) was used to guide the drilling of the CBT and MC trajectory (Fig. 3), respectively. While pushing the navigation tool firmly to the lamina, initial screw holes were made (Fig. 2). After a K-wire was inserted in the canal, the MySpine® guide was removed. Prior to the subsequent screw insertion, an appropriate cannulated tap was used, which was guided by the K-wire. However, TT screw implantation was based on the freehand technique whereby its trajectory was oriented towards the anatomic axis of the pedicle (Fig. 3). Medacta Universal Screw Technology (M.U.S.T., Medacta International SA, Castel San Pietro, Switzerland) was used for all pedicle screws. In addition, the TT screws were augmented by using 1.25 mL PMMA-based bone cement after screw insertion. To prevent possible impacts of cementation leakage on the non-cemented screw of the contralateral side, TT screw augmentation was first performed after the CBT or MC screw was tested.

### Biomechanical testing

#### Static test

**Fig. 4:**
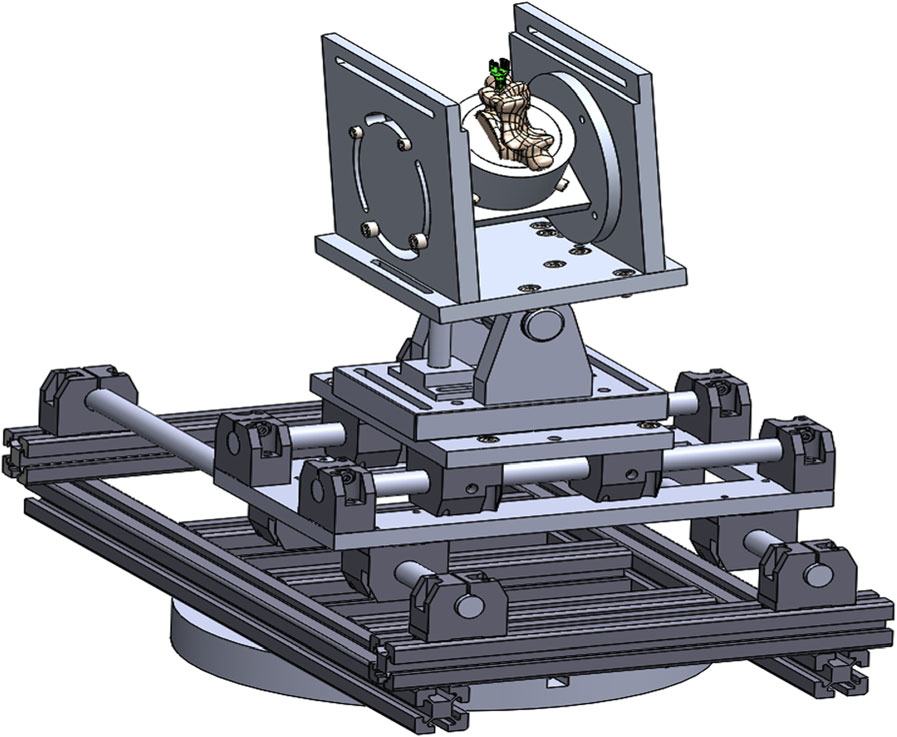
Pull-out test setup

After implantation, the appropriate subgroup was tested. Therefore, the embedded specimen was mounted and oriented properly in the customized pull-out test setup (Fig. 4). As the inserted screw was aligned with the loading axis of the servo-pneumatic uniaxial testing machine (Type 2082/000, DYNA-MESS Prüfmaschinen GmbH, Aachen, Germany), the setup could ensure the longitudinal extraction of the implant. The pull-out test was performed by using a testing speed of 5 mm/min following ASTM F543–17 [3]. During the test, force and displacement were recorded. The test procedure was stopped when the screw was released from its vertebra, which was indicated by a 50% decrease in peak force. Group A was tested randomly. By contrast, MC screws of group B were always tested first to avoid adverse effects on the screw’s fixation strength caused by TT screw augmentation.

#### Dynamic test

**Fig. 5:**
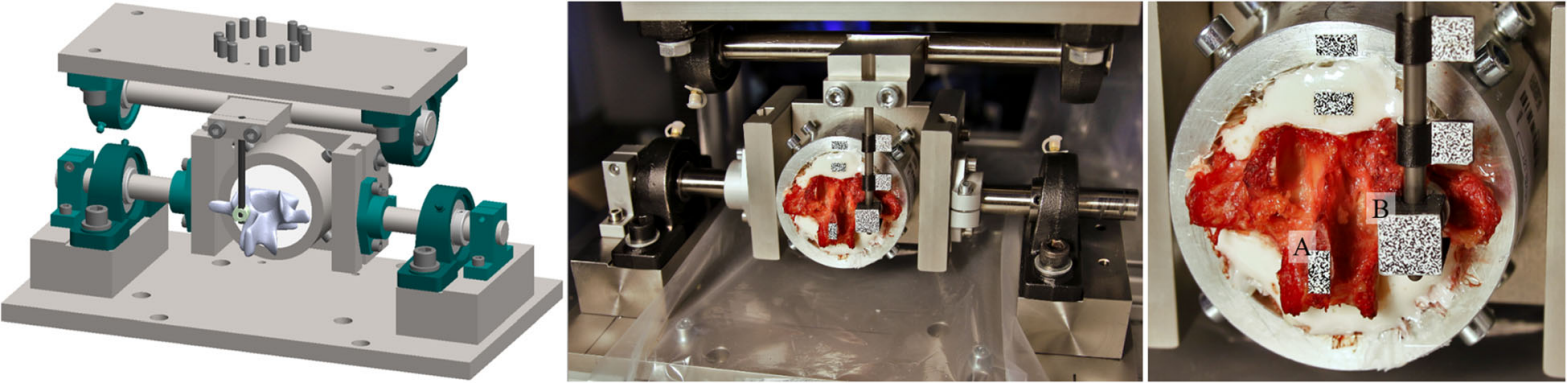
ASTM F1717–18 test setup; left: 3D CAD construction, middle: experimental setup, right: marker setup

The dynamic testing procedure, especially the experimental setup, was based on ASTM F1717–18 [4]. The test setup used (Fig. 5) enabled standardized test conditions for each vertebra despite its anatomical conditions. However, in contrast to ASTM F1717–18 [4], only one screw-rod system was evaluated. Moreover, the cranial vertebra was replaced by a customized lever arm which was directly attached to a longitudinal rod. As in the pull-out tests, group A was tested in a random order, while the MC screws were always tested first in group B. The screws underwent cyclical loading at a rate of 1 Hz for 10,000 cycles using the servo-pneumatic uniaxial testing machine (Type 2082/000, DYNA-MESS Prüfmaschinen GmbH, Aachen, Germany). The test procedure started with a preloading of 10 N (compressive force). Then a mean compressive force of 60 N was applied and cyclic testing was performed with an amplitude of ±50 N. The failure criterion was defined as a doubling of the initial screw displacement resulting from the mean compressive force (60 N) at the first cycle. In case the initial screw displacement was too high, the test was stopped when the upper and the lower setup component came into contact. For defined cycles, the relative motion of the screw-bone interface was detected by using specific optical markers (Fig. 5) and a digital image correlation system (Q400, LIMESS Messtechnik und Software GmbH, Krefeld, Germany). The displacement analysis was performed in Excel 2013 (Microsoft Corporation, Redmond, WA, USA) and Matlab R2019a (The MathWorks Inc., Natick, MA, USA). After the dynamic testing, the screws were pulled out axially, as described above, to determine the remaining postfatigue fixation strength of the screws.

### Statistical analyses

The datasets were compared descriptively using Excel 2013 (Microsoft Corporation, Redmond, WA, USA). The data of each vertebra are listed in the supplement 1–3. Due to the small number of cases, the nonparametric Wilcoxon signed rank test was performed. SPSS 24.0 (IBM, Armonk, NY, USA) was used for all statistical analyses. The statistical significance was set at *p* < 0.05. Most data were expressed as mean ± standard deviation (SD).

## Results

### Specimens

A total of ten body donors (four females, six males) were analysed (Table 1). The specimens of group A showed a mean age of 86.4 years and mean BMD of 0.839 ± 0.104 g/cm^2^. Group B had a mean age of 86.2 years and a mean BMD of 0.820 ± 0.145 g/cm^2^. All values did not differ significantly.

**Table 1:** Specimens’ baseline characteristics

Cadaver ID	Group	Sex	Age in years	Body mass in kg	Bone mineral density in g/cm^**2**^
1	A	m	82	82	0.906
2	A	m	78	60	0.774
3	B	m	90	69	1043
4	B	m	97	58	0.743
5	A	f	85	30	0.985
6	A	f	80	70	0.730
7	B	f	96	41	0.653
8	A	m	86	50	0.798
9	B	m	92	61	0.819
10	B	f	77	86	0.842

Group: A - CBT/MC, B - MC/TT; Sex: f - female, m - male

Both groups (CBT/MC, MC/TT) consisted of five body donors of five lumbar vertebrae each. While L1 and L3 of each cadaver were tested statically, L2, L4, and L5 were tested under dynamic testing conditions. However, L5 were only examined in group B.

### Static test

**Fig. 6:**
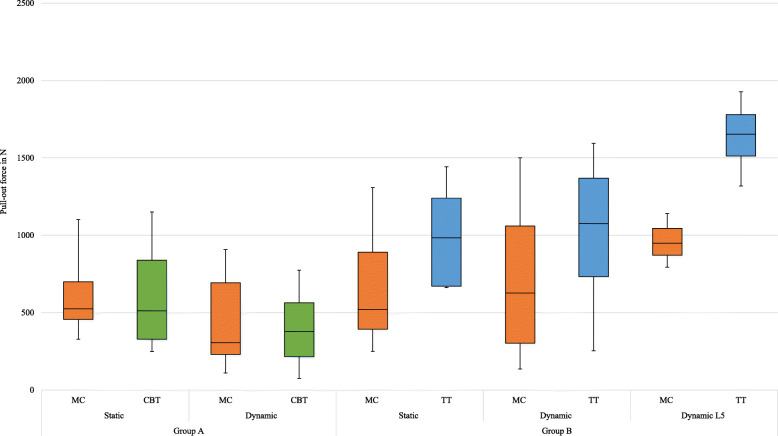
Box plots representing pull-out forces of the appropriate pedicle screws (total) in consideration of the testing conditions

In group A, 18 out of 20 screws were successfully pulled out. MC screw data were obtained from five L2 and three L4, whereas all ten CBT screws could be tested without any problems. In group B, one L4 had to be excluded because of its anatomical deformations. Hence, only 16 out of 18 screws could successfully pulled out. All MC screws could be tested in five L2 and four L4. By contrast, cement-augmented TT screws data were obtained from only four L2 and three L4. The group-specific mean pull-out forces of the different screws are shown in Table 2 and plotted in Fig. 6. All values did not differ significantly in consideration of *n* ≥ 5.

**Table 2:** Screw’s group-specific mean pull-out forces in consideration of the testing conditions

	Pull-out force in N					
	Group A			Group B		
	CBT	MC	p	TT	MC	p
**Static test**						
Total	587.9 ± 309.1	603.8 ± 227.7	0.327	986.8 ± 302.7	691.0 ± 375.2	0.063
L2	581.3 ± 307.7	649.2 ± 266.4	0.345	1003.5 ± 341.4	559.7 ± 382.6	0.068
L4	594.5 ± 310.4	528.2 ± 104.1	0.593	964.6 ± 239.9	855.1 ± 292.1	1000
**Dynamic test**						
Total	401.2 ± 261.4	449.4 ± 298.9	0.068	990.2 ± 451.9	714.5 ± 488.0	0.499
L1	424.7 ± 349.5	339.1 ± 285.9	0.180	1193.0 ± 311.7	928.5 ± 579.3	0.465
L3	377.8 ± 115.6	633.2 ± 219.1	0.180	736.8 ± 471.7	500.4 ± 221.4	0.593
L5	–	–	–	1637.9 ± 222.8	960.2 ± 141.5	0.109

Static test: pull-out; Dynamic test: fatigue testing + pull-out

### Dynamic test

In group A, only one out of ten MC screws failed prematurely due to loosening after 1500 cycles (L3, cadaver ID: 05). By contrast, five CBT screws from three L1 (cadaver ID: 05, 06, 08) and two L3 (cadaver ID: 05, 08) did not reach the scheduled 10,000 cycles. They already loosened after 64, 450, 120, 26 and 260 cycles, respectively. In group B, one L3 (cadaver ID: 09) had to be excluded caused by its anatomical deformations. Thus, only nine vertebrae could be examined. No signs of failure or loosening were observed according to the cement-augmented TT screws. Three MC screws reached the failure criterion already after 26 cycles (L1, cadaver ID: 07), 1510 cycles (L1, cadaver ID: 04), and 2144 cycles (L3, cadaver ID: 04), respectively. Four more L5 were tested on sponsor’s demand. All screws successfully resisted cyclic loading.

**Fig. 7:**
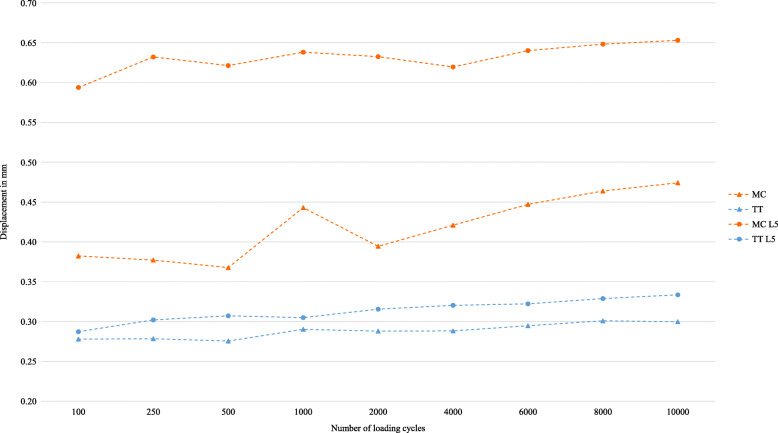
Mean displacement of screw head relative to its vertebra - CBT screw vs. MC screw

**Fig. 8:**
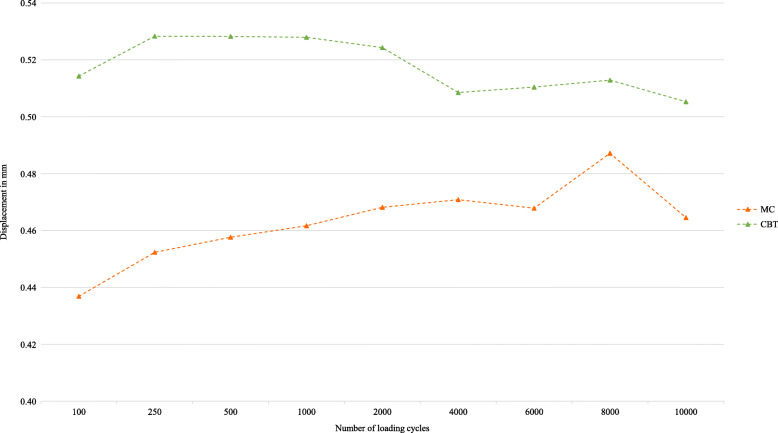
Mean displacement of screw head relative to its vertebra - cement-augmented TT screw vs. MC screw

The mean displacement was only analysed of those screws that reached total 10,000 cycles. In group A, the mean displacement between screw and bone according to the defined cycles was always lower in the MC screws (*n* = 9) compared to the CBT screws (*n* = 5) (Fig. 7). In group B, the cement-augmented TT screws (*n* = 9) showed always a lower mean displacement compared to the MC screws (*n* = 6) (Fig. 8) The same behaviour can be seen in the additionally tested L5 (*n* = 4) (Fig. 8). Because of the different numbers of vertebrae, there is no individual comparison.

The subsequent pull-out tests were only performed if no screw loosening occurred during the dynamic testing. As some vertebrae were breached, only four CBT screws and eight MC screws could be analysed. The pull-out tests (Table 2) showed that the remaining mean fixation strength of the MC screws (449.4 ± 298.9 N) was slightly higher compared to the mean pull-out force of the CBT screws (401.2 ± 261.4 N) (Fig. 6). As one vertebra had to be excluded and three MC screws loosened early, only six MC screws were pulled out, whereas nine TT screws could be tested. The MC screws (714.5 ± 488.0 N) were inferior to the cement-augmented TT screws (990.2 ± 451.9 N) concerning mean postfatigue fixation strength (Fig. 6). This fact was also confirmed by the tested L5. Here, the cement-augmented TT screws (*n* = 4) with a mean fixation strength of 1637.9 ± 222.8 N were superior to the MC screws (*n* = 3), which showed a mean pull-out force of 960.2 ± 141.5 N. But in a direct comparison of the vertebra-specific screws, MC screws showed two times (L1, cadaver ID: 09; L3, cadaver ID: 10) a higher mean pull-out force than the cement-augmented TT screws.

## Discussion

There are many different instrumentation techniques of the lumbar spine at present. However, there is still no clear consensus regarding the optimal screw design and screw trajectory, enhancing screw’s fixation strength significantly. Especially the treatment of osteoporotic bone is still challenging. As osteoporosis causes more loss of cancellous bone than cortical bone, special measures are needed. In the literature, cement-augmented pedicle screws are described as gold standard in osteoporotic spine instrumentation. Numerous biomechanical studies have demonstrated an increased pull-out strength of these screws [7, 8, 12, 16, 39]. Apart from that, good functional outcomes and low revision rates have been proven in clinical middle- and long-term studies [2, 5, 10, 11, 33]. However, cement augmentation is associated with several disadvantages such as the risk of cement leakage and subsequent embolism, exothermic properties or complications during the removal of the screws in case of revision [17, 19, 37]. Therefore, alternative techniques for the treatment of bones of compromised quality are necessary. In consideration of the required surgical demands, the CBT screw seems to be a promising approach. This is described as an attractive technique due to its less invasiveness. Furthermore, these thinner and shorter screws are characterized by their extensive contact with the solid cortical bone in contrast to TT pedicle screws. Thus, fixation strength rises, which is of particular relevance in osteoporotic bone. Santoni et al. [35] first reported the superiority of CBT screws in osteoporotic cadaveric lumbar spines. In their report, CBT screws demonstrated a 30% greater uniaxial pull-out strength and an equivalent strength against toggle loading as compared to non-augmented TT screws. Baluch et al. [6] also compared the fixation strength of these screws. But they simulated more physiological conditions using cyclical loading and subsequent orthogonal screw pull-out. Their results also demonstrated the superior resistance of CBT screws. As there is no in vivo biomechanical study reporting on the mechanical behaviour of the CBT trajectory, Matsukawa et al. [27] evaluated the insertional torque using the CBT and TT fixation approach, respectively. The comparison of both techniques showed a significant difference in the mean maximum insertional torque in favour of the CBT screws. Within the scope of another study of Matsukawa et al. [28], a finite element analysis was performed. The results show a 26.4% greater mean pull-out strength, a mean 27.8% higher resistance to cephalocaudal loading, and 140.2% stronger stiffness to mediolateral loading than non-augmented TT screws. However, Wray et al. [41] reported equivalent mechanical fixation properties of both approaches in their cadaveric biomechanical study including pull-out and toggling testing. Contrary results were achieved by Akpolat et al. [1], who stated that non-augmented TT screws had a better fatigue performance compared to CBT screws in vertebrae of compromised bone quality. As the use of bone cement during posterior instrumentation of the osteoporotic spine represents the gold standard, augmented TT screws were compared to possible alternatives within this study. Moreover, this study was focused on screw size, which was also done by Matsukawa et al. [30]. They analysed the ideal screw size for optimal fixation to significantly enhance screw’s fixation strength. As mechanical stress is dependent on the dimension of the bone-screw interface, there is a higher risk of loosening with short pedicle screws. To reduce this risk, Matsukawa et al. [30] suggested the use of longer cortical screws to improve vertebral load transmission and to decrease mechanical stress. Finally, their finite element study demonstrated biomechanical superiority of a long trajectory with maximum cortical purchase. Therefore, the “long CBT” or MC screw, which is directed towards a more anterior position of the vertebral body compared to the original CBT, is recommended. Ideally, the CBT screw should have a diameter larger than 5.5 mm and a length longer than 35 mm (standard size) [30]. To the best of our knowledge, no studies concerning the biomechanical behavior of MC screws have previously been published. For this reason, our study was aimed to evaluate this approach compared to the original CBT and the gold standard used in osteoporotic spine instrumentation. However, reaching the correct trajectory is challenging for surgeons as this narrow screw path has to be created in the denser bone. Moreover, there are fewer anatomical landmarks available within the limited operative field. Apart from high-level surgical skills, intraoperative fluoroscopic support is needed to enhance accuracy and safety. In this context, the use of a patient-specific screw placement guide including a preplanned screw trajectory has been considered as a promising approach [20–22, 24, 31, 34]. Farshad et al. [15] demonstrated in a randomized cadaveric study that the guided pedicle screw placement using MySpine® (Medacta International SA, Castel San Pietro, Switzerland) was superior in terms of faster instrumentation time, higher accuracy, and reduced radiation exposure compared to freehand fluoroscopically controlled pedicle screw placement. Moreover, this tool is characterized by its minimal invasiveness compared to conventional techniques. In this study, the MySpine® tool was used to ensure accurate cortical screw placement that crucially affects screw’s biomechanical properties. To analyse screws’ biomechanical behaviour, fatigue and pull-out testing were performed. In contrast to the often used pull-out test, the fatigue test setup provides a more clinically relevant failure scenario including more meaningful data [35]. Therefore, both groups were separated into two subgroups each, which were tested statically (pull-out) and dynamically (fatigue testing and ensuing pull-out), respectively.

In group A, both screw types had no significant differences concerning the static pull-out tests (Table 2). MC screws’ mean pull-out strength was only 2.7% higher than that of the CBT screws. However, this test procedure does not reflect physiological testing conditions. The dynamic comparison showed major differences both in cyclic loading and the ensuing pull-out test. Here, the CBT screws loosened early five times more frequently than the MC screws that failed only once. The CBT screw loosening always occurred within the first 500 cycles, which indicates inferior fixation. Consequently, it can be assumed that the larger length of the MC screw resulted in a better anchorage, which is proven by its comparably significant gain in screw’s stability. Similar results are demonstrated by Matsukawa et al. [30]. The ensuing pull-out tests also showed the superiority of the MC screws. Their higher mean failure loads (+ 12.1%) showed that MC screws resisted longer physiological loading than CBT screws did. This was due to MC screws’ design and screws’ trajectory, which allows more anchorage within the cortical bone. The displacement evaluation also substantiated the fact of CBT screws’ earlier loosening and higher range of motion during physiological cyclic loading.

In group B, the MC screws were compared to the cement-augmented TT screws mostly used for osteoporotic spine instrumentation. As expected, the pull-out tests showed higher mean failure loads of the TT screws both in the static (+ 42.8%) and dynamic (+ 38.6%) testing conditions. Weiser et al. [40] have even demonstrated that cement augmentation of osteoporotic bone can lead to an increase in failure load by approximately 52%. That can be attributed to the higher screw-bone purchase caused by the cement augmentation filling the porous bone. However, MC screws’ mean pull-out force of 691.0 N (static) or 714.5 N (dynamic) provides sufficient stability. Additionally, screws revision is possible without difficulty, whereas vertebrae mostly breach during pull-out of the cement-augmented TT screws. Moreover, screw’s solid augmentation results in a lower mean displacement compared to MC screws. The same can be observed based on the additionally tested L5. The tests also showed the superiority of the cement-augmented TT screws in both mean displacement and pull-out forces. But in a direct comparison of the L5, the MC screw showed once a lower range of motion during dynamic testing.

However, this study has some limitations. The limited number of specimens may be of concern. Apart from that, the varying sample sizes of the individual groups should be critically reviewed. Therefore, a more extensive evaluation using equal sample sizes is desirable. Furthermore, the position of the embedded vertebrae is not physiological, nevertheless a standardized procedure regarding literature. Finally, for biomechanical testing, only cadaveric specimens were used.

## Conclusion

The cement-augmented TT pedicle screws had the best fatigue performance as well as the highest pull-out forces in lumbar vertebrae of compromised bone quality. However, MC screws represent a promising alternative in case of reduced bone quality compared to the CBT screws as they showed substantially better results. Proving if MC trajectory is superior, a comparative study of non-augmented TT screws and MC screws is already planned. Especially, dynamic tests should be performed as they provide a more clinically relevant failure scenario.

## Supplementary Information

**Additional file 1: Supplement 1–3.** The data of each vertebra.

## Data Availability

The datasets used and/or analysed during the current study are available from the corresponding author on reasonable request.

## Abbreviations

BMD: Bone mineral density
CBT: Cortical bone trajectory
CT: Computed tomography
DXA: Dual-energy X-ray absorptiometry
L: Lumbar vertebra
MC: Midline cortical
PMMA: Polymethylmethacrylate
SD: Standard deviation
TT: Traditional trajectory
